# Coupling neutron reflectivity with cell-free protein synthesis to probe membrane protein structure in supported bilayers

**DOI:** 10.1038/s41598-017-03472-8

**Published:** 2017-06-13

**Authors:** Thomas Soranzo, Donald K. Martin, Jean-Luc Lenormand, Erik B. Watkins

**Affiliations:** 1 Synthelis SAS, 5 avenue du Grand Sablon, 38700 La Tronche, France; 2 University Grenoble Alpes, TheREx, TIMC IMAG/CNRS, UMR 5525, F-38000 Grenoble, France; 3 University Grenoble Alpes, SyNaBi, TIMC IMAG/CNRS, UMR 5525, F-38000 Grenoble, France; 4 Institut Laue-Langevin, 6 rue Jules Horowitz, BP 156, 38042 Grenoble, Cedex 9 France; 5 MPA-11: Materials Synthesis and Integrated Devices, Los Alamos National Laboratory, Los Alamos, New Mexico 87545 USA

## Abstract

The structure of the p7 viroporin, an oligomeric membrane protein ion channel involved in the assembly and release of the hepatitis C virus, was determined from proteins expressed and inserted directly into supported model lipid membranes using cell-free protein expression. Cell-free protein expression allowed (*i* ) high protein concentration in the membrane, (*ii* ) control of the protein’s isotopic constitution, and (*iii* ) control over the lipid environment available to the protein. Here, we used cell-free protein synthesis to directly incorporate the hepatitis C virus (HCV) p7 protein into supported lipid bilayers formed from physiologically relevant lipids (POPC or asolectin) for both direct structural measurements using neutron reflectivity (NR) and conductance measurements using electrical impedance spectroscopy (EIS). We report that HCV p7 from genotype 1a strain H77 adopts a conical shape within lipid bilayers and forms a viroporin upon oligomerization, confirmed by EIS conductance measurements. This combination of techniques represents a novel approach to the study of membrane proteins and, through the use of selective deuteration of particular amino acids to enhance neutron scattering contrast, has the promise to become a powerful tool for characterizing the protein conformation in physiologically relevant environments and for the development of biosensor applications.

## Introduction

Proteins incorporated into lipid bilayer cell membranes are involved in a variety of cellular processes including signaling, energy conversion and transport^1^. Membrane protein dysfunction is correlated with a wide range of diseases and they represent important drug targets for the pharmaceutical industry. However, studies of membrane protein associated disorders and development of new therapeutic approaches are hindered by the difficulty in obtaining atomically resolved structures of these macromolecules and determination of their function when incorporated into lipid bilayers. For example, the major bottle-neck for determining membrane protein structures using x-ray crystallography is difficulty in growing suitable crystals. Circumventing this issue, nuclear magnetic resonance (NMR) spectroscopy, electron microscopy (EM), and small angle neutron and x-ray scattering techniques have proven extremely valuable.

The majority of the early examples of crystallized membrane proteins were isolated from tissues where they were naturally present at high levels^1–3^. However, most therapeutically relevant targets are not found as abundantly in tissue and require protein expression to produce sufficient quantities to enable high resolution structural characterization. Although it is an effective means to produce soluble proteins, recombinant protein expression has limited applicability to membrane proteins because hydrophobic surface residues promote protein aggregation and subsequent cytotoxicity. Removing hydrophobic regions of the protein may be used to enhance solubility and alleviate aggregation but results in incomplete protein structures. An alternative approach is to reconstitute complete proteins in lipid or surfactant environments. Detergents are frequently used to supplement the role of lipids to stabilize membrane proteins, but the relationship between membrane lipids and transmembrane proteins is essential for their structure, function and stability^4–6^. Thus, determination of membrane protein structure in biologically relevant lipid environments is essential.

Planar lipid bilayers provide convenient model membrane systems that allow multiple measurement techniques to be applied to the study of membrane proteins. In the absence of large extracellular domains, membrane proteins may be incorporated into lipid bilayers formed directly on solid supports^7, 8^. However, in some cases the proximity of the solid support can result in detrimental interactions leading to an inability to incorporate proteins or to protein denaturation at the surface. To alleviate these interactions, a variety of membrane systems have been engineered to provide a hydrated space between the bilayer and the underlying support including the use of hydrophilic polymer cushions, tethered lipids, and tethered proteins^9, 10^. The incorporation of proteins into a membrane or the tethering of proteins to a surface is usually achieved through either interaction with a detergent solubilized protein solution^11^ or the fusion of proteoliposomes^12^. There are many challenges involved with such detergent solubilization and reconstitution approaches including a trial and error process to determine an effective detergent species (or mixture of species) and difficulties maintaining protein stability and function throughout the process^13^. Further, these methods rely on the detergent micelles or proteoliposomes reaching thermodynamic equilibrium with the bilayer, which limits the quantity of protein that can be incorporated. Cell-free protein synthesis offers an alternative approach for the incorporation of large amounts of membrane proteins in lipid environments. First, it is a well-established method for recombinant membrane protein expression at a qualitative and a quantitative level^14–16^. Second, when performed in contact with lipid bilayers, membrane protein insertion happens in a co-translational manner directly into the bilayer and is not limited by thermodynamic equilibrium constraints^17–19^. This approach is particularly useful for structural studies using neutron reflectometry (NR), which is a surface sensitive characterization technique widely employed in the study of supported lipid bilayers and to the study of lipid protein interactions^20, 21^. Previous NR studies have utilized a range of membrane systems including tethered lipid bilayers, multilamellar bilayer stacks, and adsorbed nanodisc films to provide suitable lipid environments to determine the structural envelope of membrane proteins^22, 23^. The cell-free approach may also be applied to insert proteins into more advanced engineered membrane systems such as these. In the case discussed here, the lack of large extracellular domains in p7 allowed a simple supported bilayer to serve as a suitable lipid environment, thereby simplifying the NR modeling and analysis. However, proteins with larger extracellular domains may benefit from coupling a cell-free protein expression approach with a tethered bilayer or other membrane systems that separate the bilayer from the underlying support.

**Figure 1:**
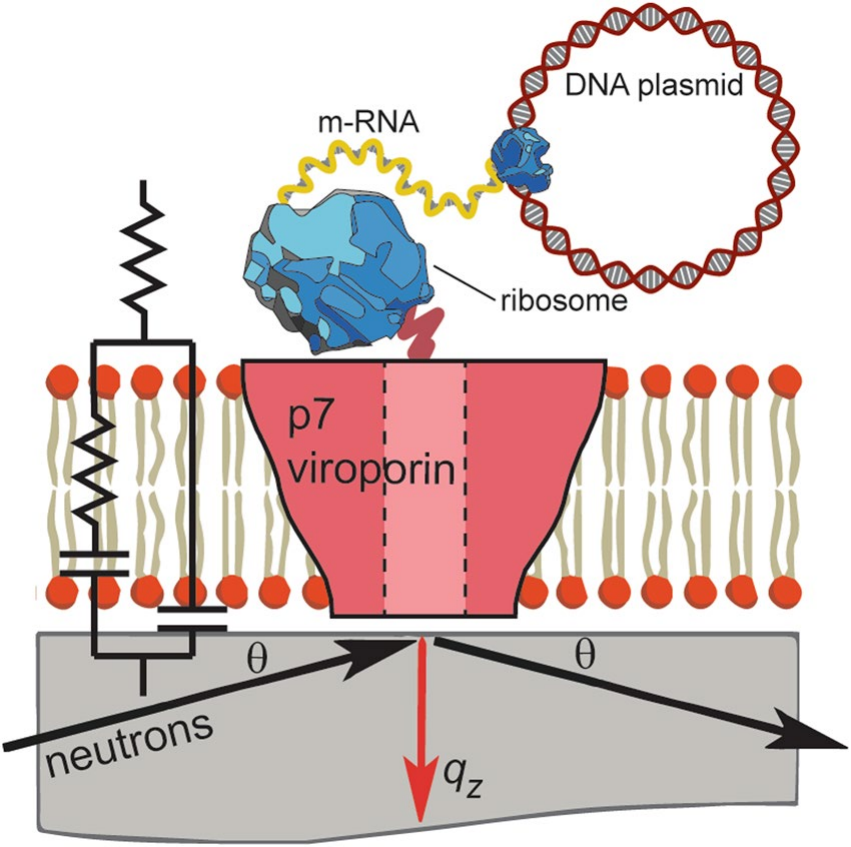
Schematic of the cell-free preparation of supported bilayers containing p7 and NR and EIS measurements (not to scale). Lipid bilayers were formed on solid supports and proteins were expressed and inserted directly into the supported membranes. For NR, membranes were formed on quartz and an incident neutron beam was transmitted through the substrate and reflected from the solid-liquid interface. The scattering vector, *q*_*z*_, is represented by the red arrow. For EIS, membranes were formed on a gold electrode used to perform conductance measurements.

In this report, we used cell-free protein synthesis to directly express a therapeutically important membrane protein target into biologically relevant single component lipid bilayers (POPC) and complex lipid mixtures (asolectin). The p7 viroporin is a transmembrane protein essential for particle assembly and release of the hepatitis C virus (HCV)^24, 25^ making it a target of interest for drug development. However, the crystallographic structure of the p7 protein has not yet been resolved and, despite EM^26^ and NMR^27–32^ characterizations, there are insufficient data on p7 structure in membranes to guide novel therapies. Here, we have taken an innovative approach to obtain structural and functional information by using deuterated amino acids during the cell-free protein synthesis to express deuterated p7directly into planar supported lipid bilayers. This approach allowed us to take advantage of enhanced isotopic contrast between the deuterated proteins and the lipids to enable neutron reflectivity measurements that provided a structural characterization of the protein incorporated in the lipid environment at nanoscale resolution (Fig. 1). Further, the system was well adapted to use electrical impedance spectroscopy to measure the transport functions of the incorporated p7 viroporin. This system enabled a large amount of p7 viroporin to be incorporated in a naturally folded configuration so as to maintain its transport functions.

## Results

### Neutron reflectivity from supported lipid bilayers

**Figure 2:**
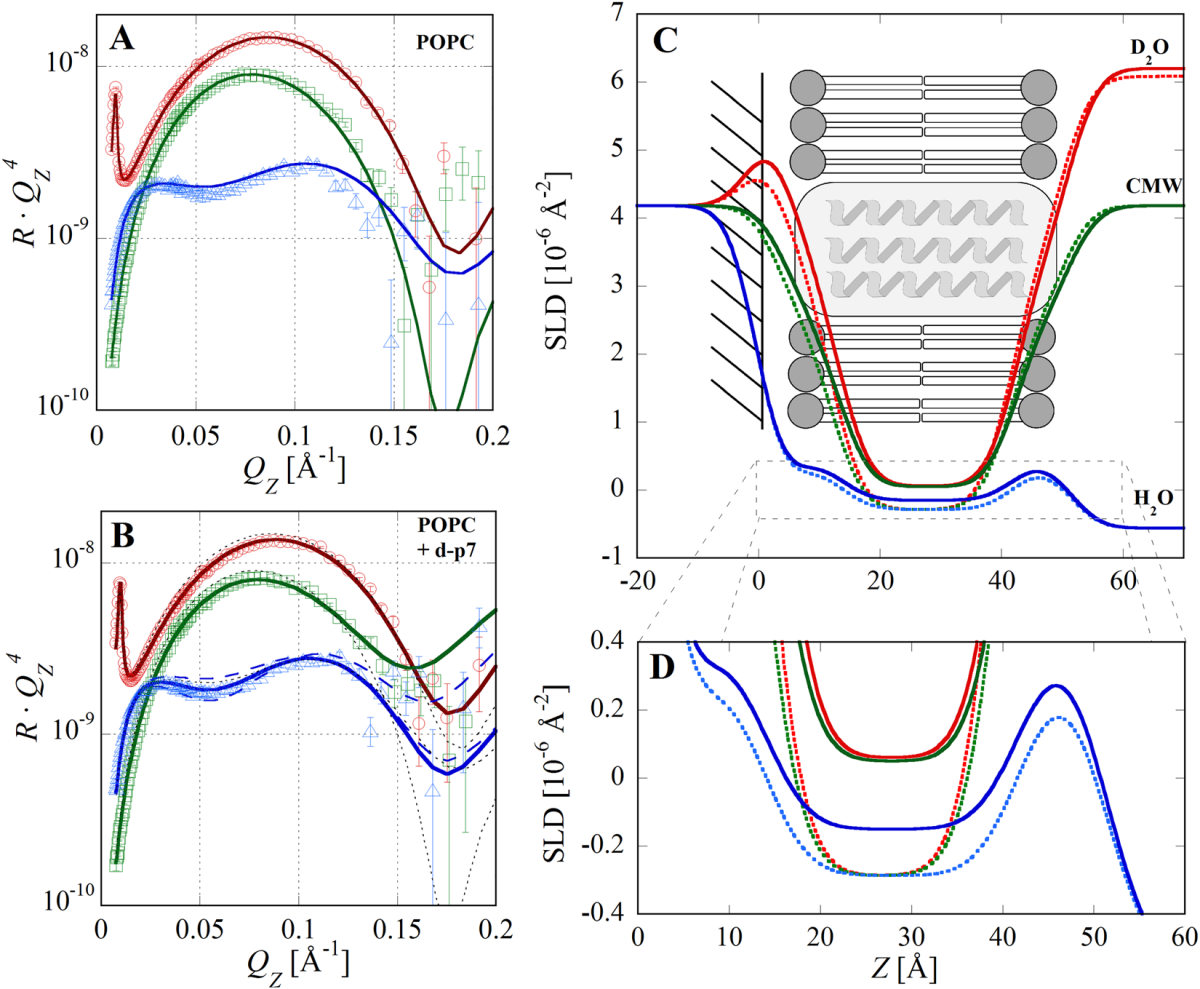
NR data and SLD profiles for POPC supported bilayers before and after d-p7 incorporation. (**A**) NR data and fits for the bilayer measured in D_2_O (red circles), CMW (green squares), and H_2_O (blue triangles). Data is presented multiplied by *q*_*z*_^4^. (**B**) Data and fits after d-p7 incorporation. For comparison, dotted lines are the fits before protein incorporation. Solid lines are fits using a cylindrical protein model with a uniform SLD distribution spanning the bilayer. Dashed lines are the best fits to the H_2_O data without protein (below) and with protein but without water penetration (above). (**C**) SLD profiles before (dotted lines) and after (solid lines) d-p7 incorporation. The schematic indicates the location of bilayer components and quartz interface (z = 0). (**D**) Magnification of the lipid tails region shows increased SLD, particularly in the H_2_O contrast, indicating incorporation of d-p7 into the bilayer.

NR data from the supported POPC bilayer was fit using the simplest possible model, which consisted of four layers describing the inner and outer head groups, the lipid tail region, and a wetting layer of water between the quartz and inner lipid leaflet. For further simplicity, the interfacial roughness between all layers was fixed to the measured roughness of the quartz substrate and the bilayer structure was constrained to be symmetric (equivalent inner and outer head group parameters). Further, the thicknesses and SLDs of the lipid heads were fixed to values obtained from high resolution x-ray diffraction measurements^33, 34^. With the constraints described, the only free parameters were the thickness of the wetting water layer and the degree of solvent penetration into the lipid heads and tails (Table SI1). This highly constrained model was capable of closely reproducing the measured data for all three measured contrasts: H_2_O, D_2_O and quartz contrast matched water (CMW) (Fig. 2A). The best fit (*χ* ^2^ = 7.7) corresponded to a full coverage bilayer with a 4.5 Å water layer between the inner head groups and the quartz substrate (Fig. 2B).

**Figure 3:**
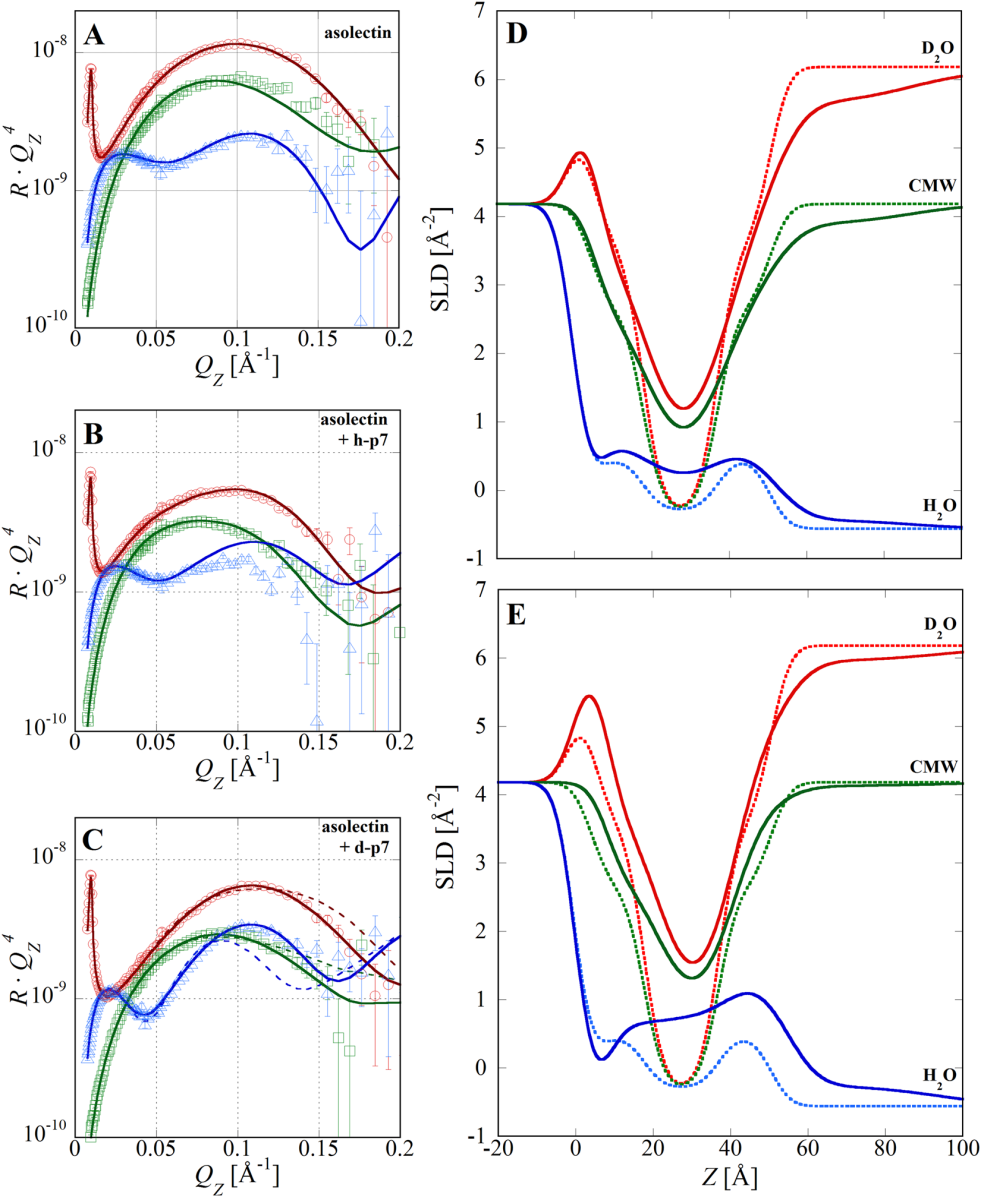
NR data and SLD profiles for asolectin bilayers before and after incorporation of either h-p7 or d-p7. (**A**) Data and fits for the bilayer measured in D_2_O (red circles), CMW (green squares), and H_2_O (blue triangles). Data is presented multiplied by *q*_*z*_^4^. (**B**) Data and fits after h-p7 incorporation using a cylindrical protein model with a uniform SLD distribution spanning the bilayer. (**C**) Data and fits after d-p7 incorporation. Dashed lines are best fits using a cylindrical protein model and solid lines are fits using a conical protein model. (**D**) SLD profiles of the asolectin bilayer before (dotted lines) and after (solid lines) h-p7 incorporation corresponding to the fits in panels A and B. The quartz interface is at z = 0. (**E**) SLD profiles before (dotted lines) and after (solid lines) d-p7 incorporation corresponding to the fits in panel A and the conical protein model fits in panel C.

A four layer model comparable to the POPC bilayer model was used to describe the supported asolectin bilayer. Again, interfacial roughness of all layers was fixed to the measured quartz roughness and the bilayer structure was constrained to be symmetric. However, due to the complex mixture of lipid constituents in asolectin there is no precise structural characterization from x-ray diffraction or other techniques to tightly constrain the parameters corresponding to the lipid tail and head group moieties. In this case, the thicknesses and SLDs of the tails and head groups were permitted to vary freely. The three contrast data sets were best fit (*χ* ^2^ = 7.1) to a model structurally similar to that of POPC (Fig. 3A–C). Both supported bilayers exhibited complete coverage and comparable total thickness. The main structural differences observed were that, relative to POPC, the asolectin tail region was significantly thinner and the head groups were thicker with a slightly higher SLD. Asolectin is a naturally extracted soy lipid mixture containing a majority of poly-unsaturated lipids with roughly equal proportions of phosphocholine (PC), phosphoethanolamine (PE), and phosphoidylinsitol (PI) head groups. Despite the lack of a precisely defined phospholipid composition, the molecular constituents of the asolectin mixture can explain the structural differences between the POPC and asolectin bilayers. Concerning the thickness of the tails, a predominance of poly-unsaturated lipid tails is likely to yield a thinner tail region relative to mono-unsaturated POPC. In the head group case, PI moieties are significantly bulkier and have higher carbon and oxygen content than PC and their significant presence in asolectin will have the tendency to increase both the head group thickness and SLD.

### Neutron reflectivity from p7 protein inserted into lipid bilayers

After cell-free expression of p7, the membrane structure was modeled as a combination of area fractions of lipid bilayer, p7 protein, and water. For simplicity, we assumed that p7 incorporation did not perturb the lipid order and all parameters corresponding to the lipid bilayer were fixed to the values obtained in the initial bilayer fits (see supplemental information﻿,Table SI1). However, the models were allowed the freedom to increase the thickness of the water layer between the substrate and the overall interfacial roughness of the membrane, which may suggest the presence of a small amount of protein protruding into the space between the bilayer and the substrate. However, models explicitly including such protein protrusions did not yield significant χ^2^ reductions to justify the added complexity and additional model parameters. Any water content in the bilayer was assumed to span the membrane with a uniform distribution. As a first approximation, the p7 protein was described as a uniform SLD cylinder spanning the membrane. The SLD of p7 was estimated by approximating the monomer volume as 9 nm^3^,based on the total of the constituent amino acid volumes, and assuming that H-D exchange with the solvent was limited to 80% of the 91 labile hydrogens due to secondary and tertiary protein structure^35, 36^. For example, the SLD of hydrogenated p7 (h-p7) was 2.56 10^−6^ Å^−2^ in D_2_O and 1.73 10^−6^ Å^−2^ in H_2_O. In the case of deuterated p7 (d-p7), the protein SLD was calculated to be 7.61 10^−6^ Å^−2^ in D_2_O and 6.77 10^−6^ Å^−2^ in H_2_O.

Due to the <5% area fraction of protein and limited contrast between h-p7 and the lipid bilayer, NR measurements of the cell-free expression of h-p7 in the presence of supported POPC bilayers did not conclusively demonstrate protein incorporation. However, when d-p7 was expressed the enhanced contrast was sufficient to detect and quantify the protein distribution in the bilayer (Fig. 2B). The best fit (*χ* ^2^ = 4.7) corresponded to a model composed of 95.3% POPC, 2.3% d-p7, and 2.5% water area fractions (Table SI2). Importantly, the presence of deuterated material in the bilayer indicates that there is minimum incorporation of the other hydrogenated components of the reaction mixture into the membrane. Parameter errors were estimated by incrementally adjusting the volume fraction of one component while allowing all other parameters to vary until the χ^2^ value of the fit increased by 10%. This approach obtained an approximate error in the volume fractions of ±1% for this system, demonstrating the presence of d-p7 in the POPC membrane. Parameter values for the bilayer roughness and interfacial water layer thickness did not vary significantly. Alternative models were also considered in which there was water penetration into the bilayer but no protein (*χ* ^2^ = 14.0) and protein incorporation but no water penetration (*χ* ^2^ = 7.9). These alternatives resulted in significantly higher *χ* ^2^ values indicating that the cell-free expression method introduced both protein and water into the bilayer. The water content can be ascribed to either defects in the membrane or water within the p7 pore. However, since NR provides only one-dimensional structural information the technique is incapable of distinguishing between these two possibilities.

Cell-free expression of p7 in the presence of asolectin membranes resulted in significantly higher protein incorporation allowing characterization of the protein distribution for both h-p7 and d-p7 expressions (Fig. 3D,E). For the incorporation of h-p7, the best fit (*χ* ^2^ = 8.2) corresponded to a model composed of 70.0% asolectin, 23.6% h-p7, and 6.4% water with an increase of the bilayer rms roughness to 6.1 Å. In order to model the data, an additional layer on the outer surface of the bilayer was also required. Extending approximately 4 nm into the water, this layer was highly solvated and had an SLD comparable to the protein (0.96 10^−6^ Å^−2^). We hypothesize that this layer corresponds to the hydrophilic hexa-histidine tag (His tag) residues attached to the synthesized protein although it is not possible to rule out a small contribution from p7 amino acids protruding outside of the bilayer. It is likely that the fits to p7 in the POPC membrane were not sensitive to this layer due to the lower quantity of incorporated protein. A similar model was used to fit the asolectin membrane containing d-p7. In this case the best fit (*χ* ^2^ = 10.0) was obtained for area fractions of 79.8% asolectin, 12.8% p7, and 7.4% water and a bilayer rms roughness of 6.2 Å. While the d-p7 volume fraction in the membrane was approximately half that of h-p7, it is unclear whether the difference is due to random variation in the cell-free expression yield or to fundamentally different expression or insertion of deuterated proteins. As in the previous case, an additional highly hydrated 3 nm layer, corresponding to the His tag, was required. For d-p7, the 3.20 10^−6^ Å^−2^ SLD of the layer was significantly higher than the SLD of the complementary layer in the h-p7 expression consistent with the presence of deuterated material originating from the cell-free protein expression and supporting the hypothesis that it corresponds to the p7 His tag.

**Figure 4:**
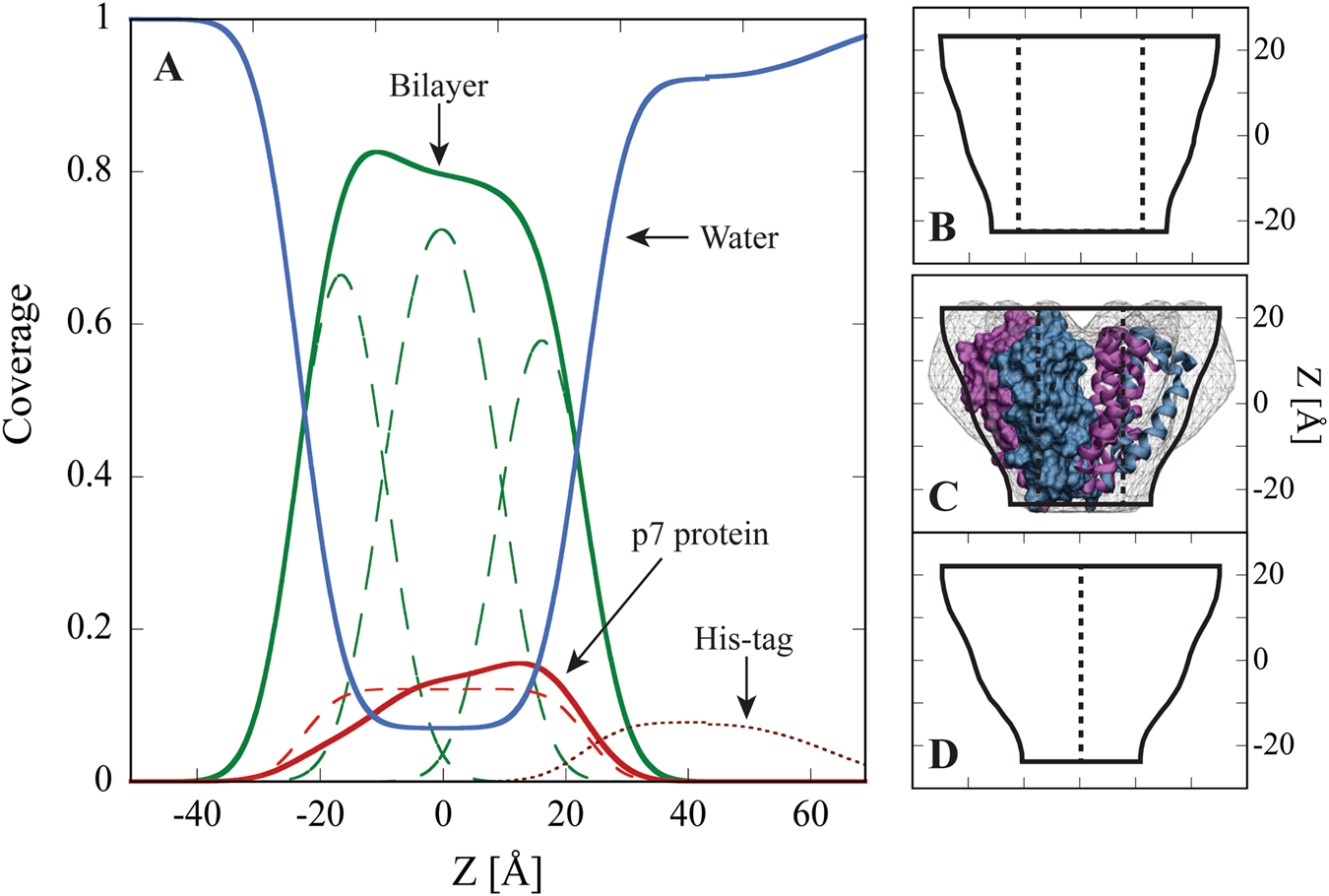
(**A**) Coverage of components within the asolectin and d-p7 membrane. The green curve represents the asolectin bilayer with dashed curves depicting the tails and head groups. Coverage of d-p7 obtained from the conical protein model is shown by the solid red line and the cylindrical protein model by the dashed red line. The dotted line is the His tag coverage and the blue line is the water distribution. Water within the bilayer may be attributed to either the protein pore volume or water filled bilayer defects. The quartz interface (at z ≈ −30) has been replaced with water for simplicity. (**B**–**D**) Assuming rotational symmetry, cross sections of the protein conformation were calculated from the conical model. Since NR is not sensitive to in-plane structure, the lateral dimension of the protein is arbitrary. Dashed lines indicate the central pore and vary depending on the fraction of water attributed to the pore volume or bilayer defects. Panel B corresponds to the protein structure in the absence of bilayer defects, panel C to 10% of the water attributed to the pore, and panel D to zero pore volume. In panel C, the conical shape obtained here is shown overlaid with the electron microscopy structure of p7 from Luik *et al*.^26^.

For the asolectin membrane containing d-p7, the combination of high neutron SLD contrast and large area coverage of protein allowed more detailed analysis of the protein distribution in the membrane. To model more complex protein structures, the constraint imposing a cylindrical protein shape was removed and the protein SLD distribution was divided into three layers. The thickness of the three layers was fixed to match the lipid head group and tail regions but the area fraction of protein in each layer was allowed to vary independently. The additional parameters described allowed the model to represent cylindrical, hourglass, biconic, or conical protein shapes. Using this model, the best fit (*χ* ^2^ = 2.7) was obtained for a truncated cone shaped protein with the wide diameter end facing away from the substrate. Protein area fractions of the distribution were 17.4% in the outer head groups, 13.0% in the lipid tails, and 6.2% in the inner head groups yielding a weighted average of 12.3%. All other model parameters were comparable to the simpler cylindrical protein distribution model but the conical protein distribution model resulted in a large reduction in the *χ* ^2^ goodness of fit. Additionally, the differences in area fraction between the three layers were significantly outside the ±2.5% calculation for the parameter error for these measurements. These results provide strong evidence for oligomerization into a conical protein shape and preferred orientation of p7 in the membrane (Fig. 4).

Control measurements were also performed where the cell-free reaction mixture was incubated with the bilayer in the absence of the DNA plasmid coding for the protein. These measurements were designed to distinguish the insertion of p7 other structural effects on the membrane induced by the reaction mixture. However, we observed that without protein expression the cell-free reaction mixture damaged and removed the lipid bilayer. This was inconsistent with the p7 expression results and it is unclear what interactions are responsible for this effect.

### Conductance measurements of p7 protein activity

**Figure 5:**
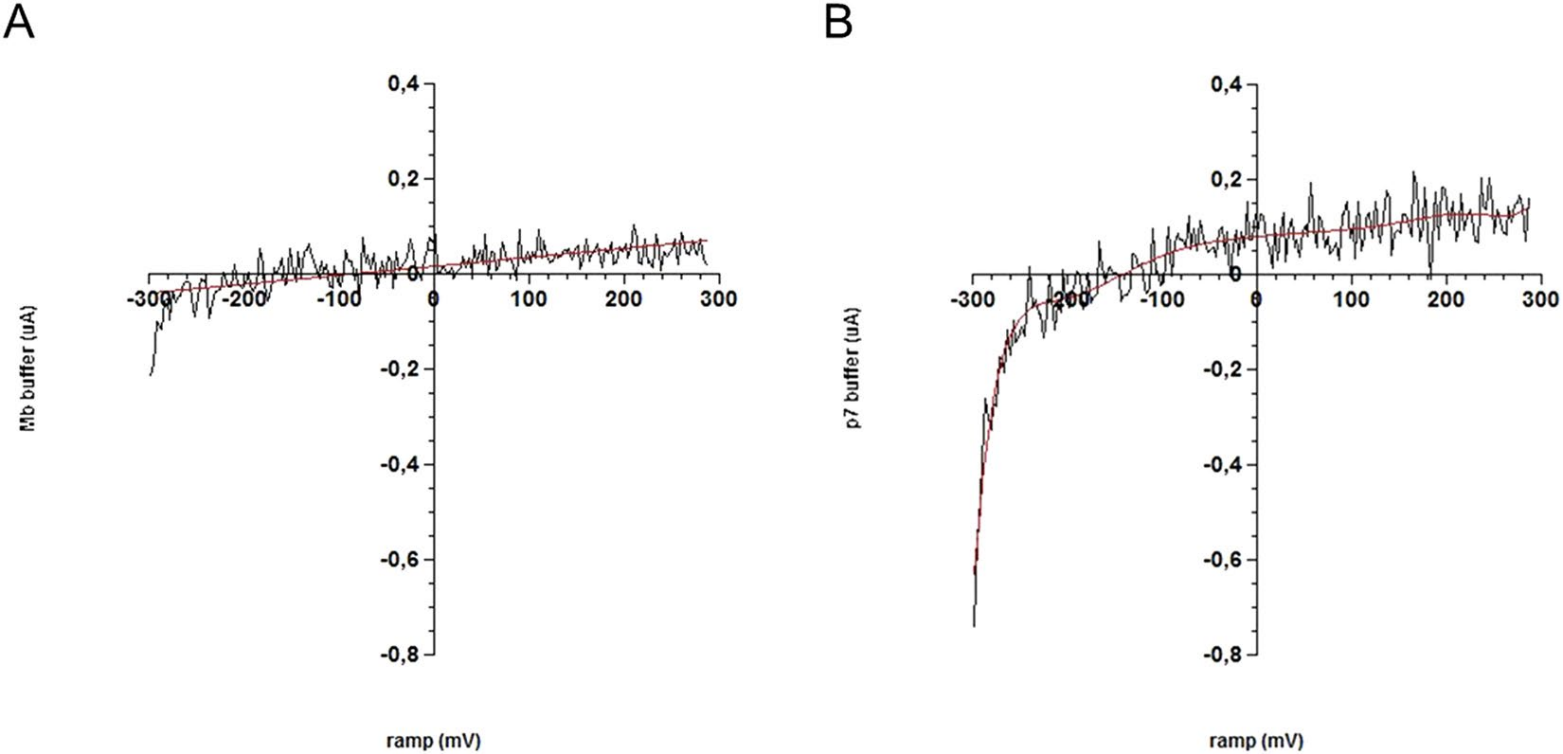
Electrophysiology recordings of an asolectin membrane on a gold electrode, before and after p7 expression. Linear regression is shown as a red line. Root mean square (rms) noise reflects membrane activity. (**A**) I/V plot of responses recorded 2 s post pulse initiation for control (**B**) I/V plot of responses recorded 2 s post pulse initiation after p7 protein expression.

To assess the function of p7 within the supported bilayer, electrophysiological recordings were performed using EIS to generate a transconductance i/v relationship under conditions mimicking those used for the NR experiments. Using an asolectin bilayer supported directly on a gold electrode, current measurements were obtained before and after p7 protein synthesis. Membranes were subjected to voltage ramps from −300 mV to +300 mV with two second duration (Fig. 5). The porin conductance is rectifying in this lipid bilayer, with a large current at negative applied potentials and a smaller current at positive applied potentials. Compared to the control condition, there is also greater p-p amplitude in the recorded signal recorded for the bilayer with incorporated p7, which is further evidence of porin activity in the bilayer. These results, coupled with the conical shape obtained from NR, strongly suggest that p7 oligomerized into a native and functional structure within the membrane. However, it is not possible to rule out the possibility that a portion of the protein exists in a monomeric state in the membrane.

Linear fits to the current yielded a conductance of 4,636 nS for the range −300 to −200 mV. Extrapolating from a 30pS single channel conductance for p7^18^, these values suggest that approximately 150,000 viroporins were incorporated in the asolectin bilayer. Using a protein area of 51.5 nm^2^, consistent with EM^26^, and the 2.1 mm^2^ electrode area 4–8 × 10^9^ proteins would be needed to match the 10-20% p7 coverage indicated by NR. Potential sources of error in the EM results, including detergent shell and stain effects, may reduce the calculated value for the number of proteins by as much as a factor of two. Additionally, small changes in the protein area induced by the different lipid environments (DHPC vs. asolectin) may also introduce error in the calculation of the number of channels. Still, large differences between the EIS and NR calculations remain which may be attributed to lower activity of p7 due to either monomeric states or to inactive channel conformation. Since macroscopic currents were measured, conductance greatly depends on channel conformation with a reduced number of open channels during the measurement consistent with lower observed activity. Viroporin conductance may also have been underestimated due to the slow ramp protocol where current may decay between voltage increments^37^. This behavior may explain the discrepancy between the quantity of incorporated protein consistent with the NR results and the lower measured conductance. Additionally, the nature of the substrate supporting the lipid bilayer may also influence protein incorporation or function. In this work, we employed substrate materials optimized for the two different techniques: single crystal quartz for NR and gold coated electrodes for electrophysiology measurements. Due to the different substrate materials, the bilayer deposition protocols were tuned to yield structurally similar and high coverage bilayers for both cases. While vesicle fusion deposition formed high coverage lipid bilayers on quartz, this method was not well suited for gold substrates and solvent exchange was used instead^38^. This approach yielded generally comparable model supported bilayer systems but the different deposition protocols and substrate materials may result in small differences in the structure and properties between the bilayers. For example, van der Waals interactions and the degree of inner head group hydration differ depending on the substrate^39^ and can impact the lateral diffusion of membrane proteins^40, 41^. It is possible that such perturbations to supported bilayer properties may impact protein integration via cell-free expression as well as decrease protein activity.

## Discussion

Cell-free expression in the presence of a supported membrane has been used to study membrane-protein interactions but has been unable to provide information about protein conformation or structure^42–44^. Here, NR coupled with cell-free expression demonstrated the expression of membrane protein (p7) directly into lipid bilayers and obtained details of the protein structure and conformation with sub-nanometer resolution (Fig. 4). Electrophysiological measurements also indicated p7 activity demonstrating that the inserted protomers oligomerize within the membrane plane and form functional proteins. The structural data obtained by NR are the first data reported for the p7 protein genotype 1a (H77 strain) and the first in a biologically relevant lipid environment. Further, use of deuterated amino acids provided increased neutron scattering contrast and unambiguously determined material in the bilayer corresponding to the synthesized protein. This approach can be further developed by expressing proteins where only specific amino acids are isotopically labeled and performing a series of neutron measurements, using either reflectivity or small angle scattering, to more precisely map out protein structures and conformations.

Most of the previously reported structural data on p7 were obtained using detergents or solvents that may perturb the protein structure. It is known that lipid environment influences p7 structure and function and this may explain discrepancies between reported p7 structures^6^. The motivation for choosing the lipids used here was that (*i*) POPC is a simple single component model system and is most representative of endoplasmic reticulum membranes^45^ where p7 is preferentially located during the viral cycle^46–50^, and (*ii*) asolectin is a more complex biologically relevant mixture, consisting of equal proportions of zwitterionic PC and anionic PS and PI head groups, which has been used previously for patch clamp characterization of p7 ion channel activity^30^. Significantly higher incorporation of p7 protein into asolectin bilayers, compared to POPC bilayers, was observed highlighting the importance of lipid composition for protein insertion. The increased integration into asolectin may be attributed to electrostatic interactions with PS and PI or the greater separation between the asolectin membrane and the underlying quartz surface.

Although high resolution crystallography of p7 has not been obtained, numerous structural studies by NMR^27, 28, 30, 31, 51^ and EM^26^ have been performed. Here, we observed that the viroporin adopts a conical shape in an asolectin bilayer (Fig. 4B–D) consistent with the helical tilt of genotype 1b (strain J4) obtained by NMR^28^ and the structure of p7 hexamer 2a (strain JFH-1) obtained by EM^26^ (Fig. 4C). The observation of a conical shape also indicates a preferred protein orientation suggesting a specific protein insertion mechanism. This finding is consistent with the cell-free expression and unidirectional insertion of connexin 43 into liposomes^52^. The His tag fused to the protein’s N-terminus extended 3 nm on one side of the lipid bilayer further indicating preferred protein orientation and suggesting that the N-terminus is located at the wide face of the conical protein. The N-terminal position of genotype 1a reported here is in agreement with the homologous structures of genotypes 2a JFH1^26, 53^ and 1b J4^51^ but conflict with the NMR structure of genotype 5a^27^. Simulations have suggested that conical p7 conformations were an artifact due to interactions with short chain DHPC lipids and that a cylindrical conformation was adopted in longer chain membranes^54^. The conical shape observed in asolectin, which has a membrane thickness more consistent with POPC than the short chained DHPC, suggests that the conical structure of p7 is not obtained only in thin membranes and that the protein adopts a conical structure in biologically relevant lipid bilayers.

## Methods

### p7 cell-free expression

The p7 protein sequence from HCV strain H77 genotype 1a was synthesized by DNA2.0. The gene was cloned directly into a pIVEX2.4d (Roche Applied Science) using NdeI/XhoI restriction sites. Cell-free expression was performed according to Soranzo *et al*.^18^. Here, mixtures of either hydrogenated or deuterated amino acids (4.08 mg/mL) were used to express isotopically labelled proteins. Expressions reactions were performed at 30 °C for 9 hrs for NR and 6 hrs for electrophysiology measurements.

### Supported lipid bilayer preparation for NR

Single crystal quartz substrates (10 × 5 × 1 cm) with 4 Å rms roughness were obtained from Mark Optics (Santa Ana, CA). Substrates were sonicated in detergent solution, rinsed with Millipore water followed by pure ethanol before being dried under a nitrogen stream. Immediately before use the substrates were UV-ozone treated for 30 minutes and assembled within solid-liquid interface cells maintained at 30 °C. Bilayers were deposited by the vesicle fusion method using either POPC (Avanti Polar Lipids) or asolectin (Sigma Aldrich). Lipid solutions were prepared in chloroform, evaporated under vacuum, and rehydrated with ultrapure water (18.2 MΩ) to a concentration of 0.5 mg/mL. Probe tip sonication was used to yield small unilamellar vesicles which were incubated with the substrate for 30 min before flushing with buffer. To incorporate p7, cell-free reaction solution was incubated with the supported lipid bilayer for 9 h at 30 °C and flushed with buffer before measurements. An HPLC pump (Knauer Smartline 1000) was used to flow solution for 10 min at a rate of 2 mL/min through the solid-liquid interface cell (volume ~2 mL) for all solution exchanges.

### Neutron reflectivity

Reflectivity, *R*, is defined as the ratio of neutrons specularly scattered from a surface to that of the incident beam and is measured as a function of the momentum transfer vector *q*_*z*_ = 4 π sin*θ*/*λ* where *θ* is the angle of incidence and *λ* is the wavelength of the beam. The resulting *R* vs *q*_*z*_ profile can be analyzed to obtain a one dimensional scattering length density (SLD) profile normal to the surface and averaged within its plane. SLD profiles derived from NR describe the density and isotopic composition of the sample as a function of depth with sub-nm resolution and can be parameterized by the thickness, SLD, and roughness of a series of layers normal to the substrate. A SLD profile corresponding to the measured data was obtained by minimizing the difference between the measured reflectivity and one calculated from a modeled SLD distribution. NR measurements were performed on FIGARO, a time of flight reflectometer at the Institut Laue-Langevin (Grenoble, France). Momentum transfer ranges of 0.008 > *q*_*z*_ > 0.2 Å^−1^ and minimum reflectivities of *R* ~ 5 × 10^−7^ were measured using wavelengths λ = 2–20 Å, two angles of incidence and a *dq*_*z*_ */q*_*z*_ resolution of 10%. Measurements were performed in 10 mM Tris pH 7.5 and 500 mM KCl buffers with three isotopic contrasts: H_2_O, D_2_O and contrast matched water (CMW), a 62:38 H_2_O:D_2_O mixture to match the quartz SLD (4.18 10^−6^ Å^−2^).

NR data were analyzed by minimizing the difference between the measured reflectivity profile and that calculated for a real space description of the membrane. Using the Parratt formalism, models consisting of a series of *n* layers of constant SLD were used to describe the SLD distribution. Error functions connecting adjacent layers were used to describe interfacial roughness. All fits were performed using custom written analysis code to enable complex constraints between layer parameters and co-refinement of the data recorded with three different SLD contrasts of water.

### Measurements of p7 conductance

For p7 conductance measurements, supported bilayers were formed by the solvent exchange method^43^. Briefly, the lipids were solubilized at 10 mg/ml in 100% ethanol and incubated for 5 min on a clean gold electrode surface (3 mm × 0.7 mm) before flushing with buffer solution (10 mM Tris pH 7.5, 500 mM KCl). The gold surface was part of a tethaPLATE system (SDx Tethered Membranes) without the self-assembled tethering monolayer. The tethaPLATE was connected to a tethaPATCH system (SDx Tethered Membranes) and a potentiostat (eDAQ, ER466) operating with a bandwidth of 100 kHz. The lipid bilayers were stimulated by voltage ramps (2 s duration) in the −300 mV to +300 mV range generated using Echem (v4.0.13, eDAQ) software. Direct incorporation of p7 into the bilayer using cell-free expression was performed using the same approach as in the NR studies. Conductance of the bilayer, with and without the incorporation of p7, was obtained from the current/voltage relationships.

## Electronic supplementary material

Supplementary information
