# Supplementary material for: Coupling neutron reflectivity with cell-free protein synthesis to probe membrane protein structure in supported bilayers
Source: Sci Rep. 2017 Jun 13;7:3399. doi: 10.1038/s41598-017-03472-8 (PMC5469739; doi:10.1038/s41598-017-03472-8)
Supplement: Supplementary file 1 — Supplementary information [file 41598_2017_3472_MOESM1_ESM.docx]

Supplementary information

**Coupling neutron reflectivity with cell-free proteins synthesis to probe membrane protein structure in supported bilayers**

Thomas Soranzo, Donald K. Martin, Jean-Luc Lenormand, & Erik B. Watkins

**Materials and methods**

*Cell-free expression of p7 with POPC and Asolectin supported bilayers*

Cell-free expression was performed using mixtures of either hydrogenated or deuterated amino acids (4.08 mg/mL) to express isotopically labelled proteins. Expressions reactions were performed at 30°C for 9hrs for NR and 6hrs for electrophysiology measurements. Because of technical constraints (namely, the duration of NR measurements), we performed cell-free expressions in the neutron cells for a longer time. The rapid depletion of energy resources and the accumulation of inhibitory by-products such as free phosphates usually lead to a short life time of the system^1^. The system used here is typically depleted within 6 hours which stops further protein synthesis^2,3^. As a result, we do not believe that significant additional protein incorporation happened in the NR relative to the electrophysiology experiments. Additional evidence reported in a similar system showed that after 1h of incubation the SLB is nearly saturated with synthesized membrane proteins (αHL-eGFP channels)^4^. After expression, a 10 mM Tris pH 7.5, 500 mM KCl buffer was used to flush the cell-free reaction mixture from the cells. This buffer was chosen to be consistent with a previous electrophysiology study of the p7 protein^5^. Moreover, we have also published results about the structure and function of p7 from genotype 1a strain H77 using the same buffer^6^. Finally, since this buffer was used to wash off the cell-free expression reaction on the bilayer, the high salinity present in the solution also served to disrupt unwanted protein-protein and protein-lipid head groups interactions.

*Cell-free expression of p7 with POPC and Asolectin liposomes*

Cell-free reaction was carried out at 30°C for 16h with gentle agitation at 400 rpm by using an *E. coli* extract and energy mix provided by Synthelis SAS in the presence of liposomes.

Liposomes were prepared using a 10 mg/ml lipid mixture of POPC or asolectin in chloroform. Chloroform was evaporated using a univapo 150H. The thin lipid film was rehydrated with diethyl pyrocarbonate treated water to obtain a 30 mg/ml lipid slurry. This solution was sonicated using a tip sonicator (Branson Digital Sonifier 250) at 20% for 5 times 30 seconds before being filtered once with a 0.22 µm PES filter.

To purify proteoliposomes, cell-free reactions were loaded on top of 3-step discontinuous sucrose gradient (60%, 30% and 5%) prepared in 50mM Hepes pH 7.5 buffer. After centrifugation at 280,000 x g for 1hr at 4°C, fractions were collected at each interface and analyzed by Western blotting using a poly histidine antibody conjugated with a horseradish peroxidase (Sigma-Aldrich) diluted at 1:10 000 in TBS-Tween buffer, 5% nonfat milk.

To assess protein integration in the lipid membrane of liposomes, proteoliposomes were subjected to alkaline extraction. Samples were diluted 1:10 in 0.1 M sodium carbonate (pH 11.5) and incubated on ice for 30 minutes before centrifugation on discontinuous sucrose gradient and analyzed as stated above.

*Electrical Impedance Spectroscopy from p7 protein inserted into lipid bilayers*

The tethaPLATE was connected to a tethaPod system (SDx Tethered Membranes) and a potentiostat (eDAQ, ER466) operating with a bandwidth of 100 kHz. The TethaPod was used to determine the low-voltage (20 mV) AC impedance spectroscopy measurement of basal membrane conduction of the lipid bilayer membrane. The TethaPod provided real-time modelling of the lipid bilayer while operating as a swept frequency ratiometric impedance spectrometer. A sequential 20 mV excitation was applied over the frequencies 1000, 500, 200, 100, 50, 20, 10, 5, 2.5, 1.25, 0.5, 0.25, and 0.125 Hz^7^.

The measured spectra were fit to the equivalent circuit model using Simplex minimisation preceded by a randomisation technique to select suitable parameters to commence the minimisation (EC-Lab, v10.44). The parameters for R_s_, R_m_, C_s_ and C_2_ were allowed to vary in order to obtain the best-fit to the spectrum for the membrane before the p7 was integrated using the cell-free expression.

*Calculation of the protein SLD*

The scattering length density of both deuterated and hydrogenated p7 was calculated for each of the isotopic compositions of the solvent by accounting for exchange of the protein’s hydrogen or deuterium atoms with the hydrogen or deuterium of the solvent. First, the protein volume was approximated based on the sum of the individual amino acid volumes in the sequence of the membrane spanning residues of the p7 monomer: ALENLVILNAASLAGTHGLVSFLVFFCFAWYLKGRWVPGAVYAFYGMWPLLLLLLALPQRAYA. The calculation was made using individual amino acid volumes obtained from Perkins et al and resulted in a monomer volume of 9nm^3 8^. Next, the number of labile hydrogen atoms in the peptide sequence, which continuously exchange with the hydrogen/deuterium of the solvent, was determined. The labile hydrogens primarily include the NH group of the main chain and hydrogens bonded to N, O, or S atoms of the side chains and, based on results of Efimova et al^9^, total 91 for the membrane spanning residues of the p7 monomer. The effect of the protein’s secondary and tertiary structure on the percent of labile hydrogrens capable of exchanging with the solvent was estimated. Using the average of the percent of exchanged hydrogens for lysozyme and β-casein given in Efimova et al^9^, we estimated the exchange of p7’s labile hydrogens with the solvent at 80%. It was assumed that all of these exchangeable hydrogens reached an equilibrium with the hydrogen/deuterium content of the solvent. Finally, after accounting for hydrogen/deuterium exchange, the total of the scattering lengths of all atoms in the monomer were totaled and divided by the monomer volume to obtain the SLD. These calculations resulted in an SLD of hydrogenated p7 (h-p7) of 2.56 10^-6^Å^-2^ in D_2_O and 1.73 10^-6^Å^-2^ in H_2_O. In the case of deuterated p7 (d-p7), the protein SLD was calculated to be 7.61 10^-6^Å^-2^ in D_2_O and 6.77 10^-6^Å^-2^ in H_2_O.

**Results**

*Additional details of the NR modelling approach*

In general, our approach was to apply the simplest possible model that was consistent with the data. For example, we assumed that p7 incorporation did not perturb the lipid order and all parameters corresponding to the lipid bilayer were fixed to the values obtained in the initial bilayer fits (Table SI1). In the first approximation, it is reasonable to assume that there is no dramatic rearrangement of the lipids upon protein insertion (i.e. a typical bilayer conformation is maintained). However, it is known that proteins can perturb lipid packing and composition in their local environment. In principle, it is possible to detect both changes in the overall membrane structure due to the protein and the protein’s conformation in the membrane as well as the more subtle changes in the lipid order. For the measurements presented here, there is a certain degree of interdependence between the parameters corresponding to the lipids and those corresponding to the proteins. This interdependence makes it impossible to independently determine the protein structure and the lipid structure. While the data could still be fit without assuming that the lipid structure is unchanged after protein insertion, imposing these constraints limits the degrees of freedom, assures that the set of models that are converged upon are physically reasonable, and we believe was the best approach to minimize uncertainty in the obtained protein structure.

Additionally, using the simplest model consistent with the data, we did not explicitly describe p7 protein protruding from the bilayer. The addition of p7 protein protrusions on either side of the bilayer were considered during the analysis but the fits did not yield a significant reduction in the χ^2^ to justify the added complexity and additional parameters of the model. Models which introduced significant protein protrusion between the substrate and the bilayer resulted in poorer fits. However, small protein protrusions (less than 5Å and with low volume fractions) can’t be ruled out. The extension of amino acids into the water phase was explicitly modeled and found to significantly improve the fits to asolectin bilayers containing p7. On the other hand, the fits to POPC bilayers containing p7 were insensitive to this layer, presumably due to the low content of protein in these membranes. In the asolectin cases, we determined that this protein extension was consistent with p7’s His tag and was too large to be attributed to protrusion of the p7 protein itself. However, it is possible that the modelled protein extension could consist of contributions from both the His tag and to regions of the p7 protein. Since the contribution from the His tag would be significantly larger, it was not possible to deconvolute the potential contribution from p7 protrusion or to describe, with any degree of certainty, extension of p7 into the water phase.

*Cell-free expression of p7 with POPC and Asolectin liposomes*

*
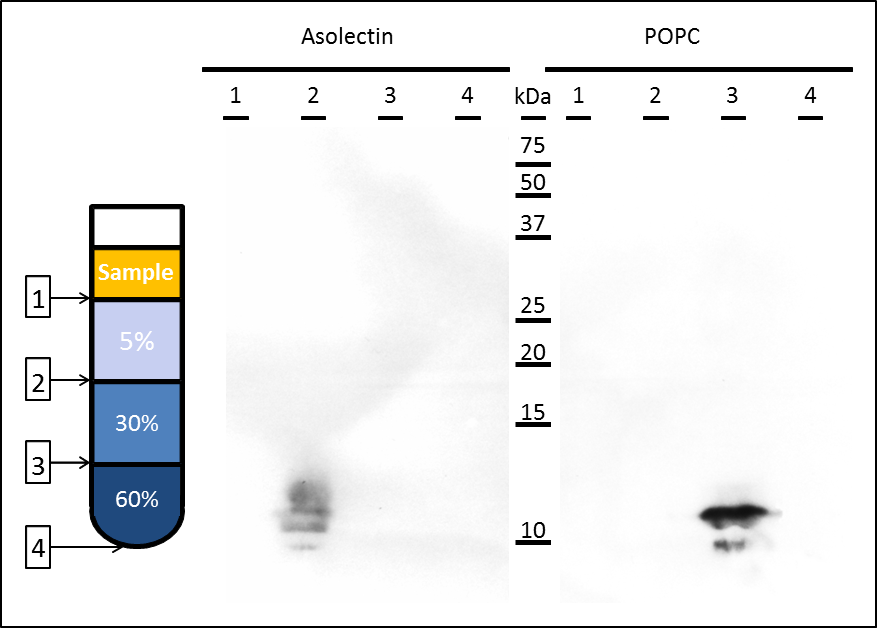
*

**Figure SI1 :** Analysis of p7 integration in asolectin and POPC liposome. Immunological detection of p7 in different fractions of a sucrose gradient after alkaline extraction and ultracentrifugation (280,000 x g for 1 h at 4 °C). Interfaces are represented as such: 1 (Sample buffer-5% sucrose), 2 (5%-30% sucrose), 3 (30%-60% sucrose) and 4 (bottom of the tube). After ultracentrifugation, p7 is found at the 5%-30% sucrose interface with asolectin lipids while with POPC, p7 is found at the 30%-60% sucrose demonstrating p7 integration in the bilayers.

Alkaline extraction is a method which has been widely used to assess if an integral membrane protein has achieved stable insertion into a lipid bilayer ^10,11^. These results suggest that the viroporin is well integrated in both types of membranes. Centrifugation using density gradient is a technique for separating particles according to their sizes, shapes and densities. POPC/p7 particles have migrated to an interface where the sucrose is more concentrated, thus these particles are larger, denser than the particles containing asolectin.

*Neutron reflectivity from p7 protein inserted into lipid bilayers*

**Table SI1 :** Fit parameters for POPC and asolectin bilayers

|  | POPC bilayer (χ^2^ = 7.7) | | |  |  | Asolectin bilayer (χ^2^ = 7.1 ) | | | |  |
| --- | --- | --- | --- | --- | --- | --- | --- | --- | --- | --- |
|  | Z  [ [Å] | SLD  [ 10^-6^Å^-2^] | Solv.  [%] | σ  [Å] |  | Z  [Å] | SLD  [ 10^-6^Å^-2^] | Solv.  [%] | σ  [Å] |  |
| Quartz | - | 4.18^†^ | - | 4.0^†^ |  | - | 4.18^†^ | - | 4.0^†^ |  |
| Water | 4.5 | 0.00^†^ | 100^†^ | 4.0^†^ |  | 4.2 | 0.00^†^ | 100^†^ | 4.0^†^ |  |
| Heads | 9.0^*^ | 1.03^*^ | 40 | 4.0^†^ |  | 13.5 | 1.34 | 46 | 4.0^†^ |  |
| Tails | 28.8^*^ | -0.29^*^ | 0 | 4.0^†^ |  | 19.4 | -0.29 | 0 | 4.0^†^ |  |
| Heads | 9.0^*^ | 1.03^*^ | 40 | 4.0^†^ |  | 13.5 | 1.34 | 46 | 4.0^†^ |  |

* Parameter fixed to values obtained from x-ray diffraction^12,13^

^†^ Parameter fixed to known values

**Table SI2 :** Fit parameters for POPC and asolectin bilayers after h-p7 and d-p7 cell-free expression. Two sets of parameters are shown, one for a cylindrical protein model and one for a conical protein model.

| Cylindrical protein models | |  |  |  | |  | | | | | |  |  | |  | | | | |  |
| --- | --- | --- | --- | --- | --- | --- | --- | --- | --- | --- | --- | --- | --- | --- | --- | --- | --- | --- | --- | --- |
|  |  | Bilayer fraction  ±0.025 | Protein fraction  ±0.025 | | Water fraction  ±0.025 | |  | Bilayer  σ  [Å] | Water Z  [Å] | His-tag layer | | | | | | | χ^2^ | |  |  |
|  |  |  |  |  |  |  |  |  |  | Z  [Å] | SLD  [ 10^-6^Å^-2^] | | | Solv.  [%] | | σ  [Å] |  |  |  |  |
| POPC +d-p7 | | 0.953^*^ | 0.022^*^ | | 0.025^*^ | |  | 4.0 | 5.0 | - | - | | | - | | - | 5.4 | |  |  |
| Asolectin +h-p7 | | 0.700 | 0.236 | | 0.064 | |  | 6.1 | 5.2 | 37.4 | 0.96 | | | 0.92 | | 6.7 | 8.8 | |  |  |
| Asolectin +d-p7 | | 0.798 | 0.128 | | 0.074 | |  | 6.2 | 9.2 | 30.0 | 3.20 | | | 88 | | 15.0 | 10.0 | |  |  |
|  | | Conical protein model  Asolectin +d-p7 | | | | | | | | | | | | | | | | |  |  |
| Heads (inner) | | 0.863 | 0.062 | | 0.075 | |  | 6.2 | 7.7 | 40.9 | 3.35 | | | 92 | | 15.0 | 2.7 | |  |  |
| Tails | | 0.794 | 0.130 | | 0.075 | |  |  |  |  |  | | |  | |  |  | |  |  |
| Heads (outer) | | 0.750 | 0.174 | | 0.075 | |  |  |  |  |  | | |  | |  |  | |  |  |

* Parameter error was < ±0.01 without the absorbed layer.

**Overlay of NR data for asolectin / p7 membranes**


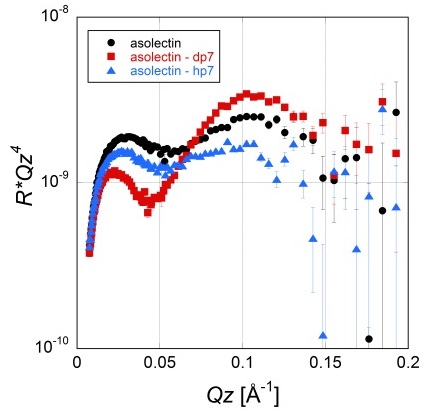

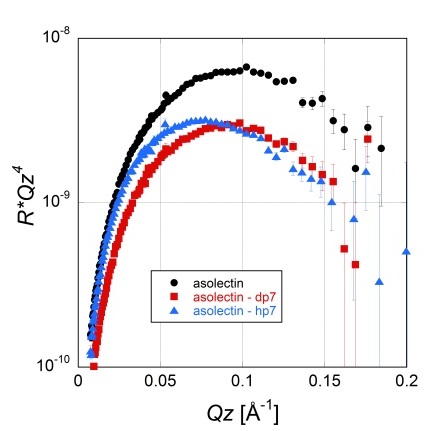

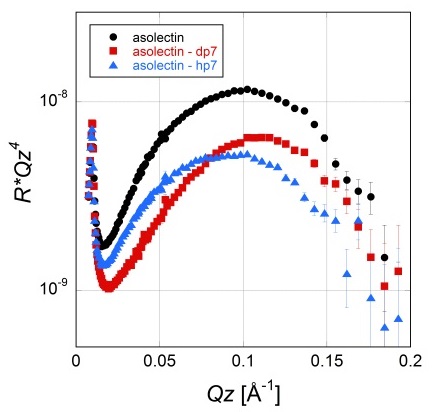


**Figure SI2 :** Comparison of NR data for asolectin bilayers before (black circles) and after insertion of d-p7 (red squares) or h-p7 (blue triangles). The left panel is data measured in H_2_O, the center panel is data measured in quartz CMW, and the right panel is data measured in D_2_O. Significant differences between the bilayer with and without protein are seen for both protein contrasts and for all three water contrasts. Data is presented multiplied by *Qz*^4^ to best show the differences in the reflected signals.

*Electrical Impedance Spectroscopy from p7 protein inserted into lipid bilayers*


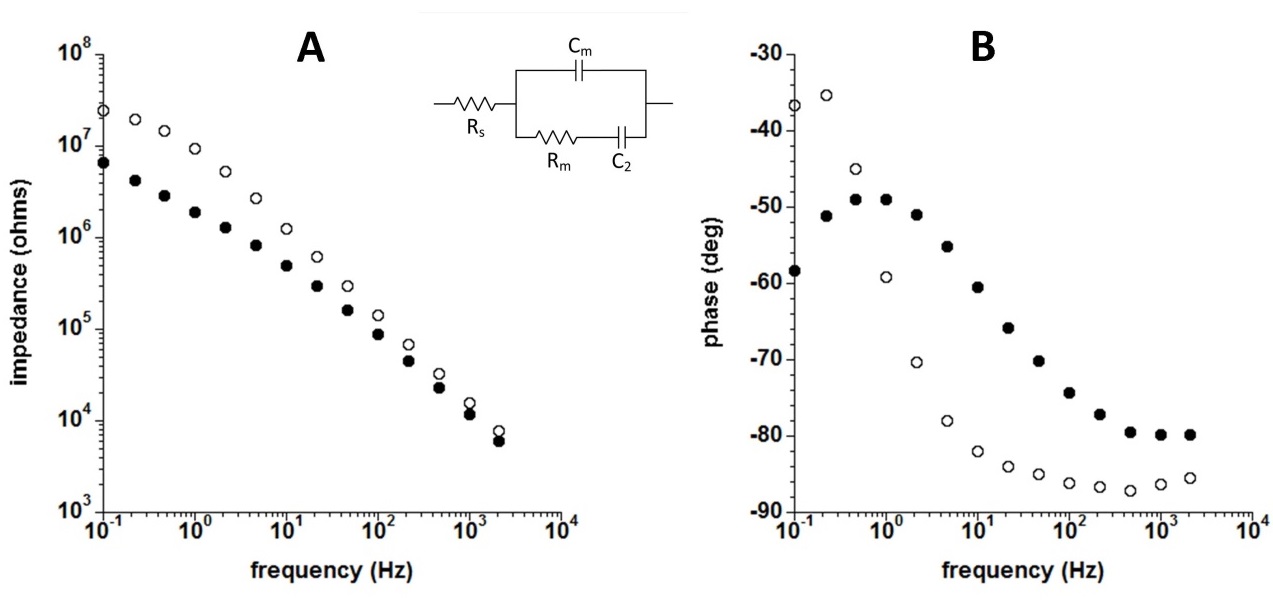


**Figure SI4 :** Electrical impedance spectroscopy (EIS) spectra of an asolectin supported lipid bilayer of 2.1 mm^2^ in area. The Bode plots are the spectra for **(A)** the impedance and **(B)** the phase before *(open circles)* and after *(closed circles)* the direct integration of p7 using the cell-free expression protocol. The inset shows the equivalent circuit model for the supported bilayer. The resistor R_s_ models the resistance of the solutions, R_m_ is the resistance of the lipid bilayer membrane, C_m_ is the capacitance of the lipid bilayer membrane and C_2_ represents the capacitance of the gold substrate and return electrode. The value for R_s_ (130 Ω) obtained from that fit was fixed for the fit of the spectrum after the integration of p7. The results of the fits to the equivalent circuit model are shown in Table SI3. The membrane before the integration of p7 was characterised by a resistance of R_m_ = 20.2 MΩ and a capacitance of C_m_ = 11.7 nF; with C_2_ = 110 nF. After the integration of p7 the membrane became more conductive, with the membrane resistance reduced to R_m_ = 0.0025 MΩ. After the integration of p7 the membrane capacitance was unchanged at C_m_ = 12.0 nF but the capacitance C_2_ needed to be modelled as a constant phase element, most likely due to a non-ideal capacitive behaviour following the integration of the p7 conducting porins combined with the capacitance of the gold substrate and return electrode. For this condition of p7 integration the fitted values of the constant phase element were Q_2_ = 183 nF.s^(α-1)^ and α_2_ = 0.505.

**Table SI3 :** Fit parameters for asolectin bilayers before and after the direct integration of p7 using the cell-free protocol. The goodness-of-fits are indicated by the *χ*^2^ and $\chi/\sqrt{N}$ values.

|  | R_m_  (MΩ) | C_m_  (nF) | C_2_  (μF) | Q_2_  (μF.s^(α-1)^) | α_2_ | R_s_  (Ω) | *χ*^2^ | $\chi/\sqrt{N}$ |
| --- | --- | --- | --- | --- | --- | --- | --- | --- |
| before p7 | 20.2 | 11.7 | 110 | - | - | 130 | 0.188 | 0.116 |
| after p7 | 0.0025 | 12.0 | - | 183 | 0.505 | 130 | 0.063 | 0.067 |

**Discussion**

*Role of lipid environment on the insertion of p7*

Our NR results indicate that protein insertion depends on the lipid environment and potentially on the intrinsic properties of the lipids. POPC is a zwitterionic lipid with a relatively large head group. It is possible that these two characteristics are responsible for the lower integration of p7 in the POPC bilayers compared to asolectin, indicating a role for steric and electrostatic forces in protein insertion. Asolectin is a lipid mixture mainly composed of lecithin (phosphatidylcholine, PC), cephalin (phosphatidylserine, PS) and phosphatidylinositol (PI) in equal proportions with 14% mono-unsaturated and 62% poly-unsaturated fatty acids. This lipid composition resulted in greater insertion of p7 proteins into supported bilayers. This increase in integration may be attributed to the presence of PS and PI, especially to their negative charges. The presence of poly-unsaturated fatty acids in the asolectin bilayer may also play a major role in protein insertion. Cis-unsaturation of the fatty tail creates a kink or bend which disrupts the in-plane packing of the tails and results in hydrophobic cavities and higher permeablity to water and other small molecules^14^. The exposed free space could drive membrane protein insertion and lead to a high proportion of p7 proteins in asolectin bilayers compared to a more tightly packed POPC bilayer. Additionally, our measurements suggest that the region corresponding to the polar heads of asolectin is thicker (13.5 Å) than that of POPC (9 Å). This thickness does not necessarily reflect larger head groups, but may indicate different spatial arrangements due to variation in the size of the hydrophobic tails. Such arrangements in asolectin could favor the insertion of the p7 protein into the membrane by alleviating steric interactions with the substrate.

*Differences in insertion of deuterated and hydrogenated p7*

In the case of p7 insertion into asolectin bilayers, a large difference in the area fraction of p7 incorporated into the bilayers was observed for the two isotopic compositions of the protein. The hydrogenated protein (h-p7) comprised 23.6% of the membrane while the deuterated protein (d-p7) only made up 12.8%. It is not immediately clear why there was such a significant difference between the amounts of protein inserted for the two different isotopic compositions. One possibility is that the difference is linked to random variation in the protein yield using the cell-free expression system employed. Another possibility is that there is a fundamental difference in either the yield of cell-free expression using deuterated amino acids or in the energetics of the insertion mechanism for deuterated proteins. Despite the common assumption in neutron scattering that isotopic substitution does not influence the structure/interactions of the system, this is an approximation at best and there are frequently significant differences in the behavior of isotopically manipulated molecules. However, we are currently unable to distinguish between these two options.

REFERENCES

1. Spirin, A. S. Continuous cell-free translation systems : history of the invention , reaction modes , and applications. (1989).

2. Sitaraman, K. *et al.* A novel cell-free protein synthesis system. *J. Biotechnol.* **110,** 257–263 (2004).

3. Kuem, J., Kim, T., Park, C., Choi, C. & Kim, D. Oxalate Enhances Protein Synthesis in Cell-Free Synthesis System Utilizing 3-Phosphoglycerate as Energy Source. *J. Biosci. Biongineering* **101,** 162–165 (2006).

4. Chalmeau, J., Monina, N., Shin, J., Vieu, C. & Noireaux, V. α-Hemolysin pore formation into a supported phospholipid bilayer using cell-free expression. *Biochim. Biophys. Acta - Biomembr.* **1808,** 271–278 (2011).

5. Montserret, R. *et al.* NMR Structure and Ion Channel Activity of the p7 Protein from Hepatitis C Virus. *J. Biol. Chem.* **285,** 31446–31461 (2010).

6. Soranzo, T. *et al.* Functional characterization of p7 viroporin from hepatitis C virus produced in a cell-free expression system. *Protein Expr. Purif.* **118,** 83–91 (2016).

7. Cranfield, C. G. *et al.* Transient potential gradients and impedance measures of tethered bilayer lipid membranes: Pore-forming peptide insertion and the effect of electroporation. *Biophys. J.* **106,** 182–189 (2014).

8. Perkins, S. J. Protein volumes and hydration effects. *Eur. J. Biochem.* **157,** 169–180 (1986).

9. Efimova, Y. M., Wierczinski, B., Haemers, S. & Well, A. A. Van. Changes in the secondary structure of proteins labeled with 125 I : CD spectroscopy and enzymatic activity studies. *J. Radioanal. Nucl. Chem.* **264,** 91–96 (2005).

10. Long, A. R., Brien, C. C. O. & Alder, N. N. The Cell-Free Integration of a Polytopic Mitochondrial Membrane Protein into Liposomes Occurs Cotranslationally and in a Lipid-Dependent Manner. *PLoS One* **7,** e46332 (2012).

11. Van der Laan, M. *et al.* Motor-free mitochondrial presequence translocase drives membrane integration of preproteins. *Nat. Cell Biol.* **9,** 1152–9 (2007).

12. Kucerka, N., Nieh, M. P. & Katsaras, J. Fluid phase lipid areas and bilayer thicknesses of commonly used phosphatidylcholines as a function of temperature. *Biochim. Biophys. Acta - Biomembr.* **1808,** 2761–2771 (2011).

13. Nagle, J. F. & Tristam-Nagle, S. Structure of lipid bilayers. *Biochim. Biophys. Acta* **1469,** 159–195 (2000).

14. Vanni, S., Hirose, H., Barelli, H., Antonny, B. & Gautier, R. A sub-nanometre view of how membrane curvature and composition modulate lipid packing and protein recruitment. *Nat. Comms* **5,** 4916 (2014).
